# Exposure to Palladium Nanoparticles Affects Serum Levels of Cytokines in Female Wistar Rats

**DOI:** 10.1371/journal.pone.0143801

**Published:** 2015-11-30

**Authors:** Ivo Iavicoli, Luca Fontana, Maddalena Corbi, Veruscka Leso, Alessandro Marinaccio, Kerstin Leopold, Roland Schindl, Alessandro Sgambato

**Affiliations:** 1 Institute of Public Health, Section of Occupational Medicine, Catholic University of the Sacred Heart, Largo Francesco Vito 1, 00168, Rome, Italy; 2 Institute of General Pathology, Catholic University of the Sacred Heart, Largo Francesco Vito 1, 00168, Rome, Italy; 3 Epidemiology Unit, Occupational Medicine Department, Research Division, Italian Workers’ Compensation Authority (INAIL), Via Alessandria, 220/E, 00198, Rome, Italy; 4 Institute of Analytical and Bioanalytical Chemistry, University of Ulm, Albert Einstein-Str. 11, 89081, Ulm, Germany; Universidad de Castilla-La Mancha, SPAIN

## Abstract

**Background:**

Information currently available on the impact of palladium on the immune system mainly derives from studies assessing the biological effects of palladium salts. However, in the last years, there has been a notable increase in occupational and environmental levels of fine and ultrafine palladium particles released from automobile catalytic converters, which may play a role in palladium sensitization. In this context, the evaluation of the possible effects exerted by palladium nanoparticles (Pd-NPs) on the immune system is essential to comprehensively assess palladium immunotoxic potential.

**Aim:**

Therefore, the aim of this study was to investigate the effects of Pd-NPs on the immune system of female Wistar rats exposed to this xenobiotic for 14 days, by assessing possible quantitative changes in a number of cytokines: IL-1α, IL-2, IL-4, IL-6, IL-10, IL-12, GM-CSF, INF-γ and TNF-α.

**Methods:**

Twenty rats were randomly divided into four exposure groups and one of control. Animals were given a single tail vein injection of vehicle (control group) and different concentrations of Pd-NPs (0.012, 0.12, 1.2 and 12 μg/kg). A multiplex biometric enzyme linked immunosorbent assay was used to evaluate cytokine serum levels.

**Results:**

The mean serum concentrations of all cytokines decreased after the administration of 0.012 μg/kg of Pd-NPs, whereas exceeded the control levels at higher exposure doses. The highest concentration of Pd-NPs (12 μg/kg) induced a significant increase of IL-1α, IL-4, IL-6, IL-10, IL-12, GM-CSF and INF-γ compared to controls.

**Discussion and Conclusions:**

These results demonstrated that Pd-NP exposure can affect the immune response of rats inducing a stimulatory action that becomes significant at the highest administered dose. Our findings did not show an imbalance between cytokines produced by CD4^+^ T helper (Th) cells 1 and 2, thus suggesting a generalized stimulation of the immune system with a simultaneous activation and polarization of the naïve T cells towards Th1 and Th2 phenotype.

## Introduction

Palladium (Pd) is a noble metal that belongs to the platinum group elements (PGEs). Over the past few decades, Pd found increasing application as an active catalyst material in modern three-way automobile catalytic converters [1, 2].

The mandatory use of these devices has resulted in a significant reduction in the emission into the atmosphere of hazardous pollutants from lean-burn engines with more than 90% of carbon monoxide, hydrocarbons, and nitrogen oxides (NOx) being converted into less harmful carbon dioxide, water and nitrogen [3–5]. Unfortunately, although these devices reduce emissions of the aforementioned pollutants, they have become a primary anthropogenic source of Pd, which is released into the environment, both in the fine and ultrafine (<100 nm) airborne particle fraction, due to the physico-chemical [6–9]. This release has inevitably increased the Pd levels in the general living and occupational environments [10–16], therefore enhancing the likelihood of human exposure to Pd particles, also in the nano-metric scale. In this emerging exposure scenario, concerns have been raised regarding the possible adverse effects Pd-NPs may exert on the human health, and particularly on the immune system of exposed subjects.

Recent evidence, in fact, demonstrated the Pd ability to induce allergic reactions in susceptible individuals generally exposed to the metal through jewellery and dental restoration contact [17–24], which could be mediated by the release of Pd ions acting as potent sensitizers [25]. Additionally, exposure to Pd-salts was demonstrated to significantly affect the production and release of different cytokines (Table 1). An increase of the interleukin (IL)-6 levels was detected in an *in vitro* skin equivalent model, consisting of human fibroblasts and keratinocytes [26]. Comparably, an enhanced secretion of IL-6 and IL-8 was observed in a three-dimensional human tissue model based on TR146 cells isolated from a squamous cell carcinoma of the buccal mucosa [27], while an and inhibiting effect on the release of IL-5, interferon (INF)-γ, and tumor necrosis factor (TNF)-α was reported in human peripheral blood mononuclear cells (PBMC) obtained from healthy male volunteers [28]. Similarly, our previous *in vivo* studies (Table 1) showed that Pd has a significant immuno-modulating effect able to alter the T-helper (Th)1/Th2 cytokine balance in Wistar rats subacutely and subchronically exposed to a Pd salt [29, 30].

**Table 1:** *In vitro* and *in vivo* studies investigating cytokine production after exposure to Pd and Pd-NPs.

**Type of study**	**Test materials**	**Experimental protocol**	**Cell lines/animal models**	**Results**	**References**
**Pd exposure**					
In vitro	Palladium with purity/composition = 99.999%	Test specimen (10 mm X 10 mm X 1 mm) was placed on human fibroblast-keratinocyte cocultures for 0.5 min, 1, 2, 3, 5, 7, 10 and 24 h	Three-dimensional cell culture system consisting of human fibroblasts and keratinocytes (Skin^2TM^ model ZK1200)	Palladium did not influence cell viability; Increased (4-fold) IL-6 levels were observed in cultures exposed to palladium.	[26]
In vitro	Palladium dichloride (PdCl_2_)	Cell cultures were exposed to 150 μl of various concentrations (0.05 mM to 50 mM) of PdCl_2_ for 24 h	Three-dimensional tissue culture model consisting of TR146 cells (from a biopsy specimen of a squamous cell carcinoma of the buccal mucosa) grown on polycarbonate filters	PdCl2 did not reduce cell viability at any concentration tested; Increased (25- to 30-fold) IL-6 levels; Increased (10- to 15-fold) IL-8 levels.	[27]
In vitro	Ammonium hexachloropalladate (NH_4_)_2_[PdCl_6_]; Ammonium tetrachloropalladate (NH_4_)_2_[PdCl_4_]; PdCl_2_.	Cell cultures were exposed to concentrations of 10^−4^–10^−7^ M of different Pd salts for 24 h	Phytohaemagglutinin (PHA) stimulated peripheral blood mononuclear cells (PBMC) obtained from 9 healthy male volunteers	(NH4)2[PdCl6], and to a lesser extent (NH4)2[PdCl4] and PdCl2, significantly inhibited IFN-γ release; Similar inhibitory effects were observed for TNF-α and IL-5 release.	[28]
In vivo	Potassium hexachloropalladate (K_2_) [PdCl_6_]	Male Wistar rats were exposed for 14 days to 1, 10, 100 and 250 ng/ml of (K_2_) [PdCl_6_]	Male Wistar rats	Increased IL-4 production; Increased IL-2 production (only at 250 ng/ml); No effects on INF-γ production.	[29]
In vivo	Potassium hexachloropalladate (K_2_) [PdCl_6_]	Male Wistar rats were exposed for 90 days to 1, 10, 100 and 250 ng/ml of (K_2_) [PdCl_6_]	Male Wistar rats	IL-2 levels were decreased up to 100 ng/ml and increased at 250 ng/ml; Increased INF-γ levels; No effect on IL-4 levels.	[30]
**Type of study**	**Type and physico-chemical properties of NPs**	**Experimental protocol**	**Cell line**	**Results**	**References**
**Pd-NPs exposure**					
In vitro	Metallic Pd-NPs with a mean size (±SD) of 10.4±2.7 nm.	Cell cultures were incubated in media alone as control or with Pd-NPs at a concentration ranging from 0.01 μg/ml to 10 μg/ml for 24 h	Lung carcinoma epithelial cell line (A549); Primary bronchial epithelial cells (PBEC).	Concentration-dependent decrease of IL-8 in the lower concentration range; Increased IL-8 levels at the highest concentration.	[31]
In vitro	Pd-NPs with 5–10 nm diameter	Cell cultures were exposed for 12 h to Pd-NPs concentrations of 10^−5^ and 10^−6^ M with and without 10 μg/ml of lipopolysaccharide (LPS)	PBMC obtained from 8 healthy female non atopic volunteers	10–5 M of Pd-NPs significantly increased the release of IFN-γ, and decreased the release of TNF-α and IL-17; No significant effects were observed on IL-5 and IL-10 release.	[32]
In vitro	Pd-NPs with 5–10 nm diameter	Cell cultures were exposed for 12 h to 10^−5^ M of Pd-NPs with and without 10 μg/ml of LPS	PBMC obtained from 12 healthy non atopic female volunteers and from 8 Pd-sensitized female volunteers	In LPS stimulated PBMC the administration of Pd-NPs significantly increased INF-γ release and reduced TNF-α release, while no significant effects were observed on IL-5 and IL-10 release.	[33]

Concerning the immunologic effects induced by Pd nanoparticles (Pd-NPs), recent *in vitro* investigations have proved the ability of such NPs to modulate the expression and release of different cytokines, although with quite different results compared to the Pd-bulk forms (Table 1). Wilkinson et al. [31] showed that the treatment of primary bronchial epithelial cells (PBEC) and lung carcinoma epithelial cell line (A549) with 0.01–10 μg/ml of Pd-NPs resulted in a concentration-dependent reduction in IL-8, in the lower concentration range, and a slight tendency towards increased levels at the highest concentration, in addition to a decrease of pro-inflammatory cytokine TNF-α in human epithelial cells. The influence of Pd-NPs (5–10 nm) on the release and expression of cytokines was also investigated in PBMC of non-atopic women exposed to 10^−5^ and 10^−6^ M of this xenobiotic [32]. In this study, Pd-NPs exerted immuno-modulatory effects enhancing the release of IFN-γ and inhibiting the secretion of TNF-α and IL-17. Similar results were also observed in PBMCs of Pd-sensitized women exposed to comparable concentrations of Pd-NPs [33].

From a public and occupational health perspective, the increasing levels of Pd in living and working environments, its well known hyper-sensitivity potential and the preliminary results concerning the ability of its nano-sized form to induce immunological alterations *in vitro*, seem to call for greater scientific efforts to define the possible immuno-toxic action of Pd-NPs in animal models. This appear an even more urgent issue of research, considering that the peculiar physico-chemical properties of materials at the nano-sized level may change their biological reactivity and potentially their harmful effects on human health [34, 35]. Therefore, the aim of the present study was to evaluate the effects of Pd-NPs on the immune system of female Wistar rats exposed to this xenobiotic for 14 days, by assessing possible quantitative changes in a number of cytokines (IL-1α, IL-2, IL-4, IL-6, IL-10, IL-12, granulocyte-macrophage colony-stimulating factor (GM-CSF), INF-γ and TNF-α).

## Materials and Methods

### Preparation and characterization of uncoated palladium nanoparticle hydrosol

As a first step, 300 μL of a freshly prepared 0.029 molar sodium borohydride solution, obtained by dissolution of 11 mg of sodium borohydride (p.a., Merck, Darmstadt, Germany) in 10 mL of ultrapure water, were diluted in 100 mL of ultrapure water. Then, 500 μL of a Pd stock standard solution (1000 mg/L, Pd(NO_3_)_2_ in 0.5 mol/L HNO_3_, Merck, Darmstadt, Germany) were added and the mixture was shaken thoroughly. The immediate color change from transparent to dark grey indicated the formation of Pd-NPs. The mixture was kept in the dark at room temperature for 12 hours to allow complete reaction.

**Fig 1:**
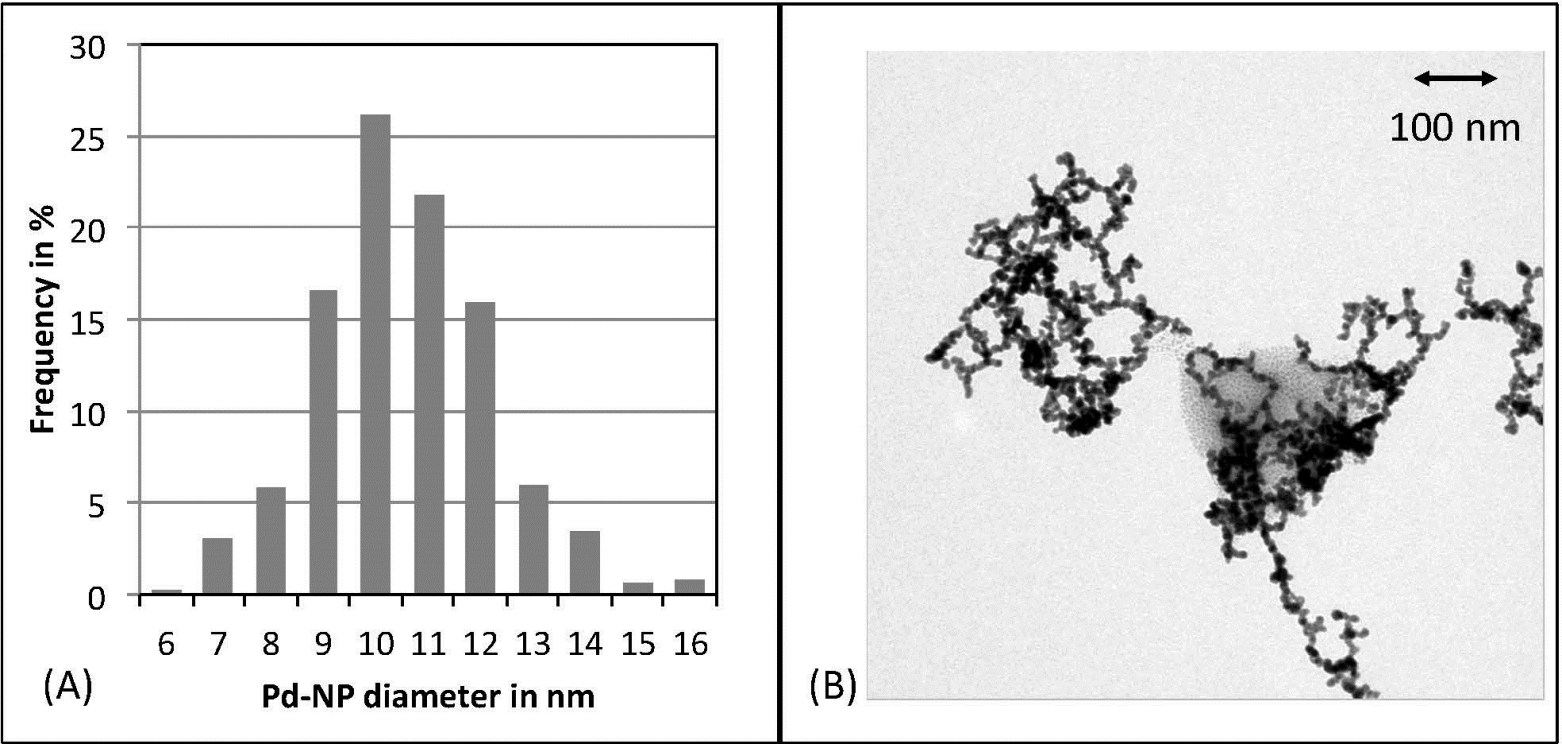
Palladium nanoparticle characterization. Mean Size distribution histogram of Pd-NPs (A) obtained from evaluation of TEM images (B) taking into account over 500 nanoparticles. The measurement of 500 individual particles depicted by TEM images revealed the size distribution of the particles to be 10 ± 6 nm.

The Pd-NPs hydrosol obtained was characterized by continuum source—graphite furnace atomic absorption spectrometry (CS-GFAAS; contrAA 600, Analytik Jena, Jena, Germany) and transmission electron microscopy (TEM; Zeiss EM 10, Carl Zeiss Microscopy GmbH, Jena, Germany) operating at 80 kV. The Pd concentration of the stock hydrosol was determined in a 100-fold dilution of the stock hydrosol in ultra pure water by means of CS-GFAAS using the spectral line at 244.791 nm. Calibration was performed in a concentration ranging from 20 to 80 μg Pd/L by applying adequate dilutions of a Pd stock standard solution (1000 mg/L, Pd(NO_3_)_2_ in 0.5 mol/L HNO_3_, traceable to Standard Reference Materials from the National Institute of Standards and Technology, Merck, Darmstadt, Germany) in 0.5 mol/L HNO_3_. This resulted in a linear calibration function with a correlation coefficient of 0.986. The stock hydrosol Pd concentration was found to be 4.71 ± 0.05 mg/L. The measurement of 500 individual particles depicted by TEM images using ImageJ software (National Institutes of Health, Bethesda, MD) revealed the size distribution of the particles to be 10 ± 6 nm (Fig 1). The hydrosol served as a stock solution for all experiments and is stable for at least 2 weeks when stored in refrigerators at 4°C. Before use, the stored Pd-NP hydrosol was homogenized by shaking vigorously. Finally, aliquots of the stock solution were diluted in ultrapure water to obtain the final concentrations used in the experiments.

### Animal husbandry

Twenty three-month-old female, pathogen-free Wistar rats were supplied by the Experimental Animal Production Plant of the Catholic University of the Sacred Heart (Rome, Italy) and allowed to acclimatize for two weeks before starting the experiment. Wistar rats are an outbred strain of albino rats employed in all fields of medical and biological research as a general multipurpose model [36]. In fact, the use of rats offers a series of advantages such as metabolic pathway similarities to humans, similar anatomical and physiological characteristics, a large database for comparative purposes [37]. In this regard, currently, the rat is definitely the species of choice for non-clinical immuno-toxicity and in particular outbred Wistar rats are often used due to their acceptable inter-animal variability [38, 39]. The animals were maintained during the entire experiment in Makrolon cages (model 1291, with overall dimensions of 425x266x185 mm and floor area of 800 cm^2^) (Tecniplast S.p.A., Buguggiate, Italy) containing a wooden dust-free bedding (model Scobis Uno, Mucedola s.r.l., Settimo Milanese, Italy), at a room temperature of 23.1°C, a relative humidity of 55% and a 12-h light/dark cycle. The animals had a mean weight of 271 ± 16 g and were fed with the solid “R” maintenance diet for rats (Altromin Rieper A. S.p.A., Vandoies, Italy). Diet and purified water were provided ad libitum to the animals. No significant changes in body weight were observed during and at the end of the experiments.

### Ethics statement

The animal study has been approved by the Ethical Committee “Commissione per la Valutazione Etica di Sperimentazioni Animali e di Correttezza della Gestione dell’”Animal Care” of the Catholic University of the Sacred Heart of Rome, Italy, under permit number 20H, and has been authorized by the Italian Ministry of Health, according to the Legislative Decree 116/92, which implemented in Italy the European Directive 86/609/EEC on laboratory animal protection. Animals used in this study were housed and treated according to Legislative Decree 116/92 guidelines and all efforts were made to minimize animal suffering.

### Animal administration and sampling of biological material

The twenty female Wistar rats were randomly divided into four exposure groups and one control group, with four rats per group. Rats were given a single injection of vehicle (control group) and different concentrations of Pd-NPs (0.012, 0.12, 1.2 and 12 μg/kg) via the tail vein. On 14^th^ day after exposure, rats were anesthetized with 0.5 mg of medetomine and 75 mg of ketamine per kg body weight. Subsequently, blood from each animal was drawn by cardiac puncture and collected in a 1.5 ml vial (Eppendorf srl, Milan, Italy). Serum samples were obtained from blood by centrifugation (3,500 rpm per 15 min) and stored at -28°C until analysis. After the blood sampling, rats were euthanized via exsanguination by cutting both the abdominal aorta and vena cava.

This particular administration route was chosen for the xenobiotic as the intravenous route of application produced a worst case scenario of 100% bioavailability. The doses used to treat animals, were selected in order to resemble possible occupational and/or environmental exposure scenarios. In fact, if we take into consideration the Pd airborne levels (highest mean level of 7.70±4.15 mg/m^3^) reported in literature for an occupational setting [40] and a human breathing rate of around 20 m^3^/day (for a man with a mean weight of 70 kg), a potential occupational exposure to Pd via inhalation corresponds to an exposure dose of 2.20 mg/kg, which is in the range of doses used in our experiments. Therefore, the higher exposure doses (1.2 and 12 mg/kg) simulated possible occupational exposure both under normal and accidental conditions during which re-exposure can occur. The lower doses (0.012 and 0.12 mg/kg) were used to investigate potential adverse effects at exposure levels closely resembling those of the general population and to establish a preliminary dose-response curve for defining the toxicological behavior of Pd-NPs [41].

### Analysis of serum cytokines

A multiplex biometric enzyme linked immunosorbent assay (ELISA)-based immunoassay, containing dyed microspheres conjugated with a monoclonal antibody specific for a target protein, was used, in accordance with the manufacturer’s instructions (Bioplex Rat Cytokine 9-Plex A panel; BioRad Inc., Hercules, CA), for the simultaneous detection and quantitation of IL-1α, IL-2, IL-4, IL-6, IL-10, IL-12, GM-CSF, INF-γ and TNF-α. Cytokine levels were determined using a Bio-Plex array reader an automated flow-based microfluidic device that uses a dual-laser fluorescent detector with real-time digital signal processing for quantitation (Bioplex, Biorad).

### Statistical methods

Statistical analysis was carried out by IBM SPSS statistics software (IBM Statistical Package for Social Sciences for Windows, Version 22.0. Armonk, New York, USA). Levels of cytokines IL-1α, IL-2, IL-4, IL-6, IL-10, IL-12, GM-CSF, INF-γ and TNF-α were measured after the four levels of exposure on day 14. The normal distribution of observed values was checked using the non-parametric Kolmogorov–Smirnov Z test and variance homogeneity was tested using the Levene test. One-way analysis of variance (ANOVA) was then performed to test the significance of differences in parameter means in the exposed and control rat groups. The Dunnett post hoc multiple comparison test was used to test the significance (*p* value Dunnett t test <0.05) of differences in values for each parameter at different exposure levels against the control group. Box-plot or linear graphs were obtained for all analyzed parameters at different exposure levels.

## Results

**Fig 2:**
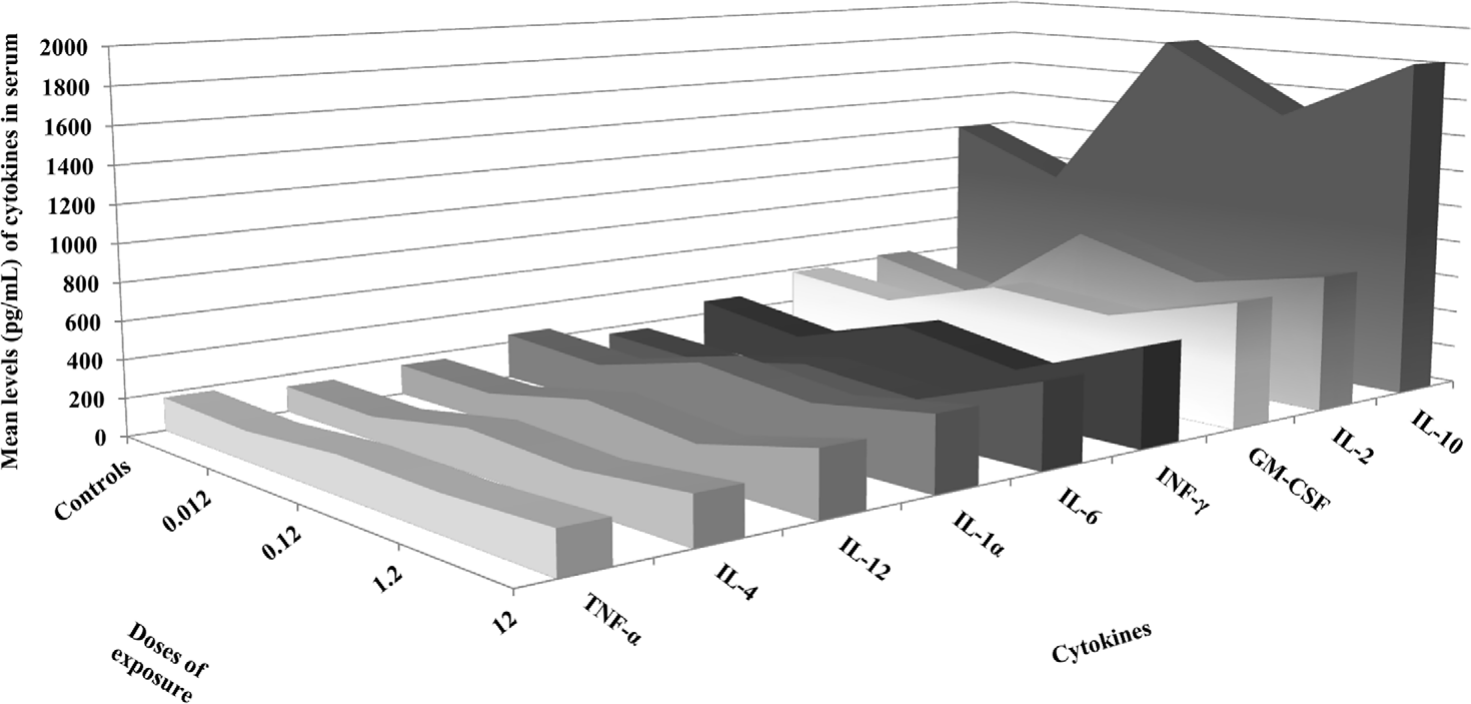
Mean serum levels of cytokines in Wistar rats exposed to Pd-NPs compared to control rats. Compared to control values, a rather particular trend was observed in all cytokine serum levels in the treated rats, with a slight decrease at the lowest exposure dose and an increase thereafter with increasing exposure doses. Indeed, the mean serum concentrations of all cytokines appeared to decrease after the administration of 0.012 μg/kg Pd-NPs, whereas their values exceeded the control levels at higher doses of exposure (0.12, 1.2 and 12 μg/kg).

**Fig 3:**
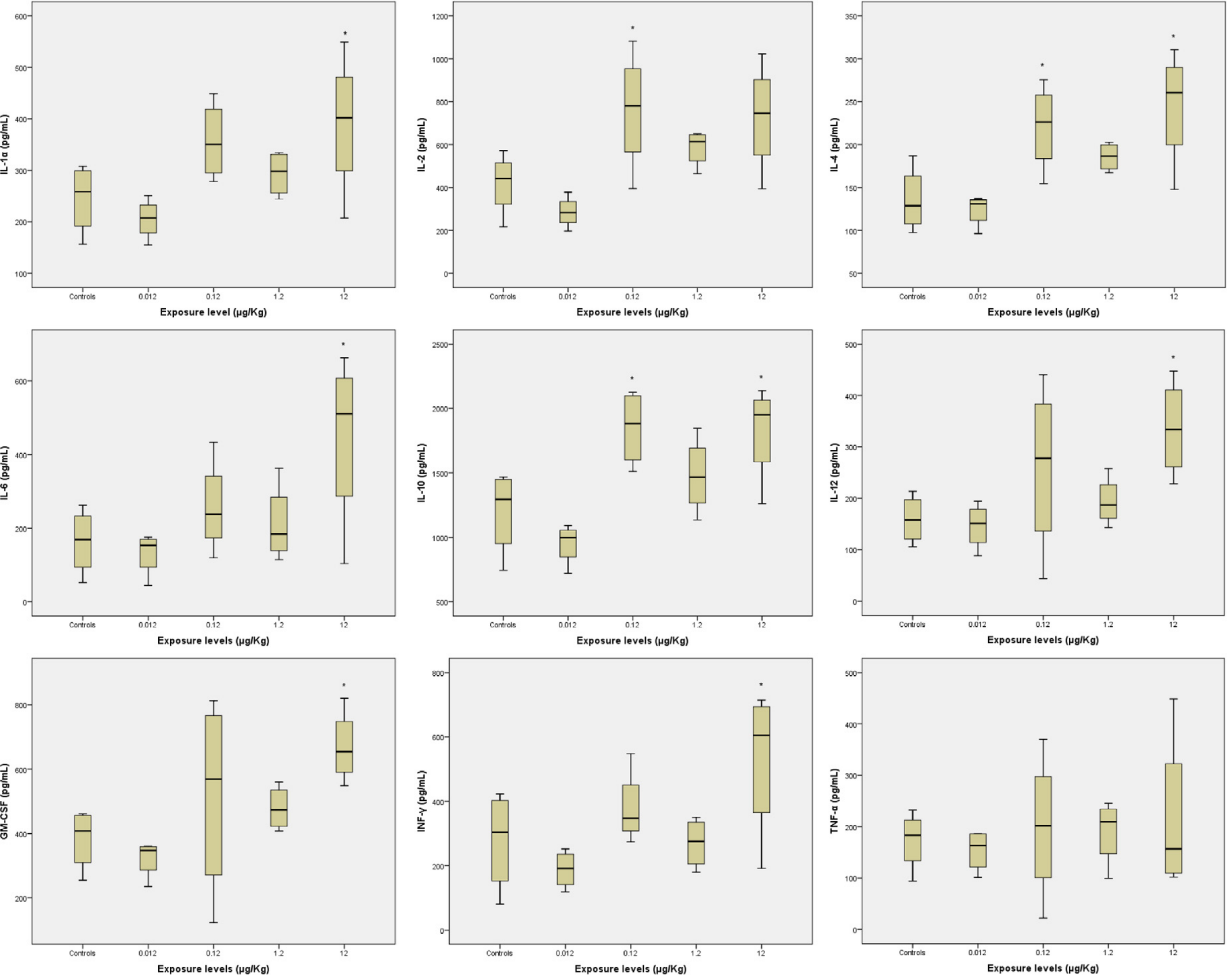
Serum levels of different cytokines in control and palladium nanoparticle exposed rats. In the exposure range from 0.12 to 1.2 μg/kg it was possible to observe a general, but not statistically significant (with the exception of IL-2, IL-4 and IL-10 at 0.12 μg/kg), increase in all cytokine serum levels, while at 12 μg/kg 7 out of 9 of the cytokines examined showed remarkable (and statistically significant) increases in serum concentrations. *Group mean significantly different from controls (p value < 0.05).

Table 2, Figs 2 and 3 and S1 Fig show serum levels of the various cytokines (IL-1α, IL-2, IL-4, IL-6, IL-10, IL-12, GM-CSF, INF-γ and TNF-α) in rats after the intravenous administration of 0.012, 0.12, 1.2 and 12 μg/Kg of Pd-NPs. The results obtained demonstrated that exposure to Pd-NPs was able to affect immune response in female Wistar rats. Indeed, each cytokine investigated showed alterations in serum concentrations compared to the control levels. The mean serum concentrations of all cytokines appeared to decrease after the administration of 0.012 μg/kg Pd-NPs, whereas their values exceeded the control levels at higher doses of exposure (0.12, 1.2 and 12 μg/kg).

**Table 2:** Mean serum levels (standard error) and statistical significance of IL-1α, IL-2, IL-4, IL-6, IL-10, IL-12, GM-CSF, INF-γ and TNF-α in control and palladium nanoparticles-exposed (0.012, 0.12, 1.2 and 12 μg/kg) female Wistar rats.

Cytokines	Number of rats	Controls	Doses of exposure (μg/kg)				*ANOVA F* test	*p* value ANOVA
			0.012	0.12	1.2	12		
**IL-1α (pg/mL)**	4	245.3 (34.4)	205.3 (19.8)	356.9 (38.3)	293.7 (22.2)	390.0* (70.2)	3.4	0.03
**IL-2 (pg/mL)**	4	417.9 (74.0)	285.2 (37.0)	759.5* (141.9)	585.3 (42.9)	727.1 (129.5)	4.5	0.01
**IL-4 (pg/mL)**	4	135.4 (19.2)	123.6 (9.3)	220.6* (25.5)	185.7 (8.4)	244.8* (34.6)	5.8	0.005
**IL-6 (pg/mL)**	4	163.0 (45.0)	131.7 (30.0)	257.0 (65.2)	211.2 (53.6)	446.8* (121.0)	3.1	0.05
**IL-10 (pg/mL)**	4	1200.1 (166.8)	952.3 (80.6)	1850.2* (148.9)	1478.9 (148.0)	1825.1* (194.1)	6.6	0.003
**IL-12 (pg/mL)**	4	158.9 (23.8)	146.3 (22.3)	259.9 (83.9)	193.7 (23.9)	335.8* (47.7)	2.8	0.06
**GM-CSF (pg/mL)**	4	383.0 (48.1)	322.7 (29.5)	518.6 (156.3)	478.5 (34.5)	669.4* (56.8)	2.8	0.06
**INF-γ (pg/mL)**	4	277.5 (78.4)	188.2 (29.5)	379.2 (59.0)	270.1 (39.3)	529.4* (118.7)	3.3	0.04
**TNF-α (pg/mL)**	4	173.2 (29.1)	153.6 (20.4)	198.9 (71.7)	190.8 (32.1)	216.1 (80.2)	0.2	0.93

ANOVA test and statistical significance (*p* value ANOVA)

Significance of the difference between mean in each exposed group and mean in the controls group

* *p* value < 0.05

The highest concentration of Pd-NPs (12 μg/kg) induced a statistically significant increase of IL-1α, IL-4, IL-6, IL-10, IL-12, GM-CSF and INF-γ compared to controls, while at the same dose of exposure the values of other cytokines, although higher than in untreated rats, did not show significant differences. A noticeable increase in IL-2, IL-4 and IL-10 serum concentrations was also observed also at 0.12 μg/kg. These results showed that the exposure to 12 μg/kg of Pd-NPs caused an important stimulatory effect on the immune system of female Wistar rats.

## Discussion

In the last few years there has been a significant increase in the Pd content of catalytic converters since this metal is cheaper and has a very high catalytic activity [2]. This enlarged Pd employment resulted in serious contamination of a number of environmental matrices with a consequent increasing likelihood of general living and occupational exposure to the metal, both in the fine and ultrafine airborne particle fractions [10–16]. Therefore, the definition of the potential health effects induced by Pd-NP exposure has become an issue of public health relevance.

Currently, most available information concerning the impact of Pd on the immune system is the result of *in vitro* and *in vivo* studies that have assessed the biological effects induced by different Pd salts [21, 26–30]. Nevertheless, the findings of these studies may not be sufficient to explain the immunotoxic potential of Pd in the nano-sized scale as potentially released from automobile catalyst abrasion and deterioration [3, 7, 9, 42–44]. The unique set of NP physico-chemical characteristics, in fact, may affect their toxico-kynetic and dynamic behavior, therefore directly or indirectly influencing the possible interactions with the immune system, in a potentially different manner compared to their bulk counterpart. This important issue prevents us to extrapolate data from Pd salts to a context of nano-sized Pd exposure [34, 35, 45, 46] and applies for a deep research on the Pd-NP toxicological profile to obtain, in turn, a more comprehensive assessment of the immunological toxicity of the metal.

Therefore, the present study aimed at evaluating the possible adverse effects of Pd-NPs on the immune system of female Wistar rats intravenously exposed to these xenobiotics through the determination of the serum levels of a series of different cytokines.

An important stimulatory action on the immune system, which becomes significant at the highest dose of treatment has been demonstrated. In fact, an overall up-ward trend, although not significant, was observed for all cytokine serum levels in the 0.12–1.2 μg/kg dose range (with the exception of significant increases detected for IL-2, IL-4 and IL-10 at 0.12 μg/kg), while at 12 μg/kg, 7 out of 9 of the cytokines examined showed significantly remarkable increases in serum concentrations (Table 2). This systemic cytokine activation supports a clear pro-inflammatory action of Pd-NPs when administered *in vivo*, which, not surprisingly, has a rather different profile compared to the immune alterations detected in previous studies exploring a variety of Pd salts. In fact, while we observed a stimulatory response on the production of IFN-γ and a slight increase (though not significant) in TNF-α, the exposure of PBMC to various Pd salts induced inhibitory effects on these cytokine secretion [28]. Moreover, the enhanced IFN-γ and IL-4 levels reported herein were not detected in our previous *in vivo* studies (sub-acute and sub-chronic exposure of Wistar rats to potassium hexachloropalladate, respectively), even if in each of these experiments some results (increased IL-4 and IL-2 production after sub-acute administration and increased IFN-γ levels following sub-chronic exposure to Pd salt) were somewhat similar to those induced by Pd-NPs [29, 30]. These quite conflicting results, further underlines the need to specifically investigate the Pd-NP interaction with the immune system which seems different from that of Pd salts, probably depending on the diverse biological reactivity determined by the peculiar chemical, optical, magnetic and structural NP properties, as previously mentioned.

Additionally, comparing our results with those obtained with Pd-NPs *in vitro*, a certain variability concerning the activated Th cell subsets and the induced cytokine profiles emerged [32, 33]. This seems an interesting topic of research, since understanding how the immune system adapts to the insults of specific xenobiotics, maybe through an excessive Th1 or Th2 responses, with different tissue damages or hypersensitivity reactions, respectively, gives the possibility to deeply understand the toxicological behavior of such NPs, therefore identifying early and specific biological alterations [47–49]. In this perspective, a clear NP induced imbalance towards a Th1 mediate immune response was recently reported in *in vitro* studies [32, 33], while our findings demonstrated a Pd-NP induced up-ward trend among all the investigated cytokines, therefore supporting a more complex and generalized inflammatory activation of the immune system in exposed animals. Overall, this suggests that the systemic cytokine activation induced by Pd-NPs *in vivo* was not related to a specific Th pattern since no imbalance was evident between commonly studied Th1 and Th2 cytokine subsets.

This may reflect the complexity of the Pd-NP interaction with *in vivo* biological systems which cannot be thoroughly resembled by *in vitro* results [50]. In fact, several *in vivo* factors such as exposure mode, penetration of physiological barriers, solubility in biological media as well as the protein corona formation, as the result of a dynamic nano-bio interaction, can dramatically change the effects of challenging the immune system with a given concentration of a specific xenobiotic [51, 52].

**Fig 4:**
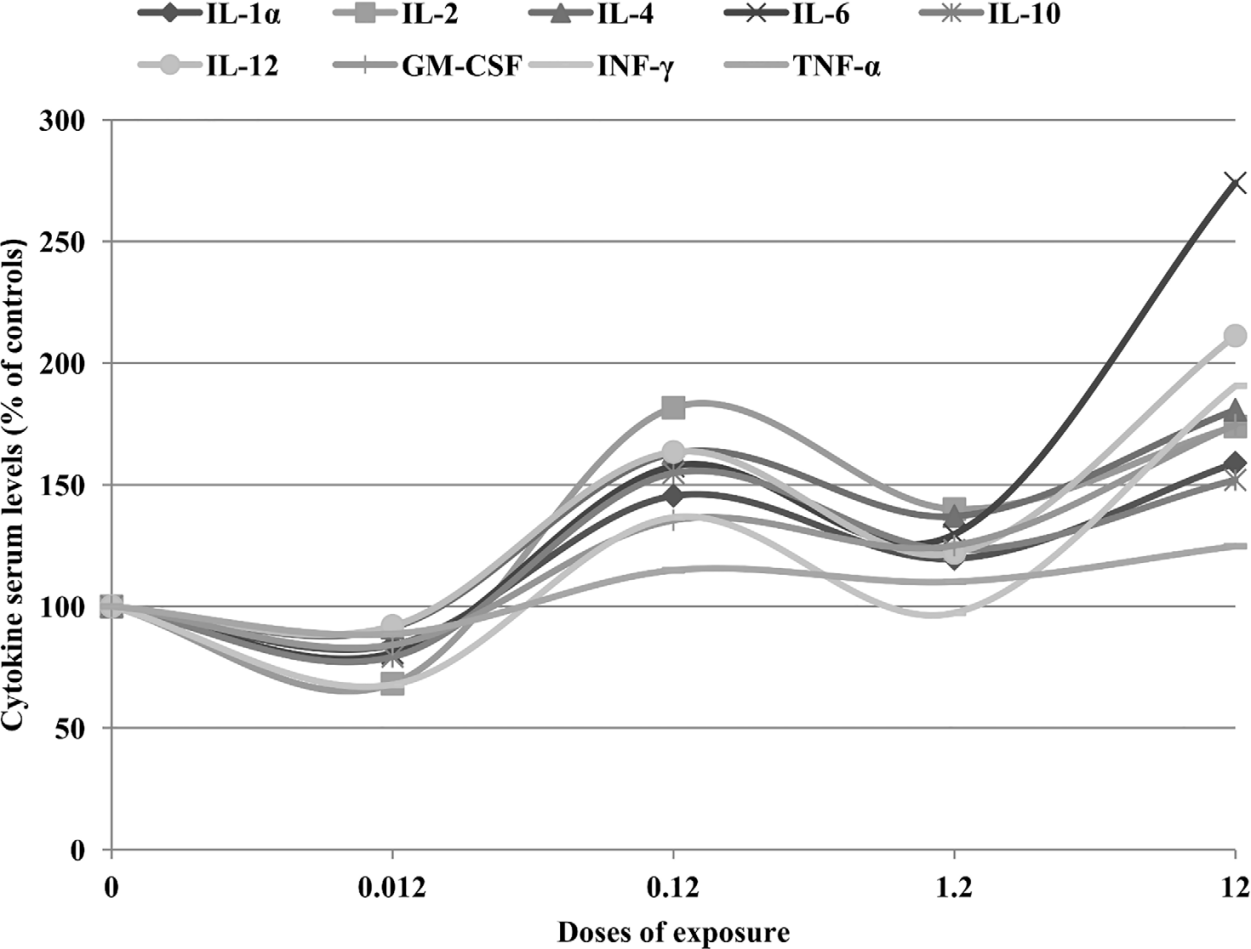
Mean serum levels of different cytokines expressed as percentage variation from control values (100%). The particular trend of the dose–response relationship observed for all cytokines (with a slight decrease at the lowest exposure dose and an increase thereafter with increasing exposure doses) would seem to suggest the presence of a hormetic phenomenon since in some cases the hormetic effects are typically graphed as a J-shaped dose response curve.

When analyzing the dose-response relationship obtained in our study, a rather particular trend was observed in all cytokine serum levels in the treated rats (Fig 2) with a slight decrease at the lowest exposure dose and an increase thereafter with increasing exposure doses. Comparably, Wilkinson et al. [31] observed a similar dose-response trend, with a decrease in the IL-8 release from PBEC and A549 cells at the lower concentration range and a slight tendency towards increased levels at the highest concentration. These dose–response relationships would seem to suggest the presence of a hormetic phenomenon since in some cases the hormetic effects are typically graphed as a J-shaped dose-response curve [53]. In fact, the term “hormesis” is used to describe dose-response curves where the response is reversed between low and high doses of a stressor (Fig 4) representing an index of biological plasticity at multiple levels of biological organization [54]. In this regard, it is possible to hypothesize that the decrease in cytokine levels determined at the lowest dose of exposure may be an adaptive compensatory process following an initial disruption in homeostasis induced by the NP chemical stress, which ultimately may induce increasing alterations in the cytokine concentrations at the higher treatment doses. Greater attention is being given to hormesis in the fields of aging and biogerontology, toxicology, pharmacology, public health and occupational medicine research, and recently this dose-response model has been shown to occur quite frequently also after exposure to different types of NPs [55]. To the best of our knowledge, this is the first time that a similar biphasic dose-response has been reported as a consequence of Pd-NP exposure. Obviously, this result should be considered with caution and further studies are needed. However, the possible presence, at low exposure levels, of effects that may be adaptive, non-adverse or even beneficial is an intriguing issue that deserves further attention particularly on account of the complex regulatory mechanisms of the immune system that favor a balance between pathogenic and protective Th cells and the crucial role that different Th subsets play in immunopathology [56].

Nevertheless, considering that this is the first *in vivo* attempt to assess the effects of Pd-NPs on the immune system and that our understanding of their immunotoxicity is still in an initial phase, the biological implications of the aforementioned alterations in serum cytokine concentrations are uncertain. However, it should be noticed that the T helper cells, producing different subsets of cytokines, are critical for a proper immune cell homeostasis and host defence, but may be also major contributors to pathology of autoimmune and inflammatory diseases [48]. Therefore, it is not possible to exclude that prolonged or repeated exposure to these xenobiotics may ultimately result in inflammatory related tissue damages, hypersensitivity or autoimmune responses triggered by the immuno-toxic alterations exerted by Pd-NPs, also in relation to possible inherent or acquired individual susceptibility factors as well as to other environmental or occupational co-exposed substances. Interestingly, extrapolated to a public health and occupational medicine perspective, the detected pro-inflammatory alterations, may act as possible early indicators of biochemical alterations induced by Pd-NPs before un-reversible organ damages or systemic diseases become manifested. These changes should be deeply verified and eventually validated as possible biomarkers to be employed in biological monitoring programs in order to assure adequate risk management strategies.

Concerning the potential mechanisms underlining Pd-NP immune effects, it is worth noting that also other types of NPs have yielded similar findings in *in vitro* and *in vivo* experiments. For example in ICR mice the administration of magnetite iron oxide (Fe_3_O_4_)-NPs induced an increase in Th1 and Th2 serum cytokine concentrations [57, 58] and titanium dioxide (TiO_2_)-NPs caused a transient release of both types of cytokines in A549 cells [59]. These results may suggest that different types of NPs may share a common molecular mechanism of action, maybe through oxidative stress reactions, that is able to exert a generalized stimulatory effect on the immune system. Oxidative stress and inflammation, in fact, are interrelated by amplification loops [60]. Pro-inflammatory cytokines, in fact, may induce a massive production of free oxygen radicals which in turn may modulate the release of inflammatory mediators by activating different transcription factors [61]. This amplification between oxidative stress and inflammation may be involved in the adverse effects caused by NPs, some of which may cause DNA damage and cell death by apoptosis [62]. However, given the limited information available on this issue, no definite conclusions can be deduced at this stage of research.

Clearly, further *in vitro* and *in vivo* studies are needed to more deeply understand the immune potential of Pd-NPs. *In vitro* experiments, in fact, may represent a valid instrument to investigate Pd-NP induced cellular changes at bio-molecular levels and to determine their underlying mechanistic processes. *In vivo* investigations, on the other side, seem necessary to define the toxico-kinetic and dynamic behavior of NPs, as well as to confirm our preliminary results also under conditions of long-term exposure resembling those experienced by the general and occupational exposed populations.

## Conclusions

Intravenous administration of Pd-NPs revealed the ability of this xenobiotic to significantly affect the immune system of Wistar rats by enhancing the serum levels of several cytokines secreted by different Th subsets. This generalized stimulatory effect was also observed in other *in vitro* and *in vivo* studies that investigated the immune potential of various NPs but differed substantially from the results of previous *in vitro* studies that evaluated the impact of Pd-NPs on the cytokine expression and release from PBMC cells. In view of the scant quantity of information currently available on the immunotoxicity of Pd-NPs, these conflicting results warrant further studies to evaluate and clarify the potentially toxic effects of Pd-NPs on the immune system and to reach a definitive understanding of this issue. This assessment appears even more urgent if we consider the increase in human exposure to Pd-UFPs and the fact that Pd salts and Pd-NPs exert different effects. Our findings differ considerably from the immunotoxic effects induced by several Pd salts in cell lines or laboratory animals, thus confirming that the unique physico–chemical properties of NPs give them a specific toxicological profile. Lastly, an evaluation of cytokine levels could be an interesting and promising biomarker for providing a more adequate assessment and management of risk with regard to nanomaterial exposure and effects [63].

## Supporting Information

S1 Fig Detail of cytokines serum levels observed in control rats and in four groups of female Wistar rats exposed to different levels of Pd-NPs. (DOCX)
