# Supplementary material for: Exposure to Palladium Nanoparticles Affects Serum Levels of Cytokines in Female Wistar Rats
Source: PLoS One. 2015 Nov 30;10(11):e0143801. doi: 10.1371/journal.pone.0143801 (PMC4664404; doi:10.1371/journal.pone.0143801)

S1A Figure Serum levels of cytokine IL-1α of control rats and of four groups of female Wistar rats exposed to different levels of palladium nanoparticles.


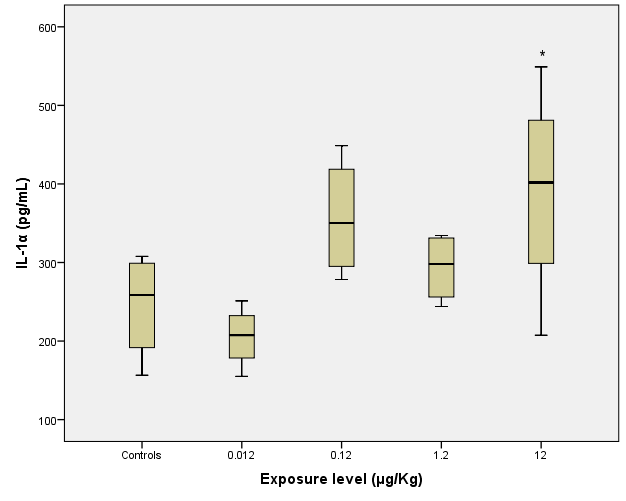


* Group mean significantly different from controls mean (p value < 0.05)

S1B Figure Serum levels of cytokine IL-2 of control rats and of four groups of female Wistar rats exposed to different levels of palladium nanoparticles.


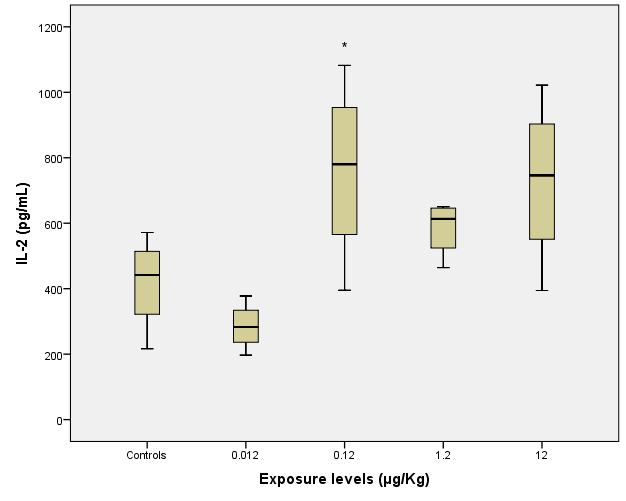


* Group mean significantly different from controls mean (p value < 0.05)

S1C Figure Serum levels of cytokine IL-4 of control rats and of four groups of female Wistar rats exposed to different levels of palladium nanoparticles.


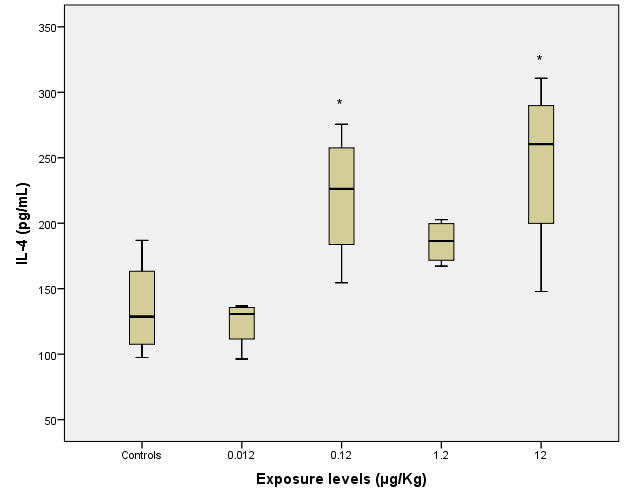


* Group mean significantly different from controls mean (p value < 0.05)

S1D Figure Serum levels of cytokine IL-6 of control rats and of four groups of female Wistar rats exposed to different levels of palladium nanoparticles.


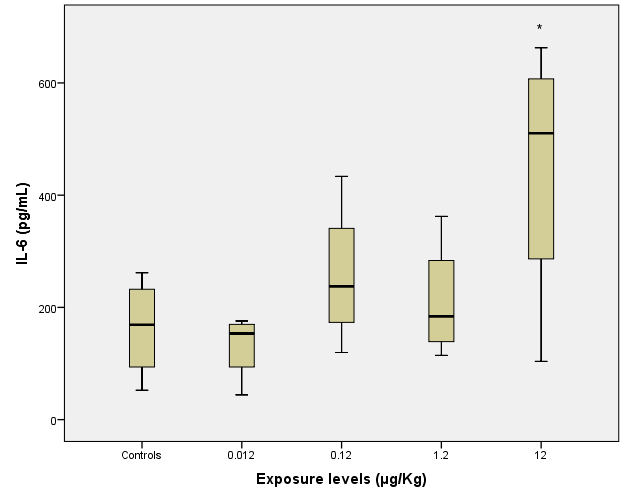


* Group mean significantly different from controls mean (p value < 0.05)

S1E Figure Serum levels of cytokine IL-10 of control rats and of four groups of female Wistar rats exposed to different levels of palladium nanoparticles.


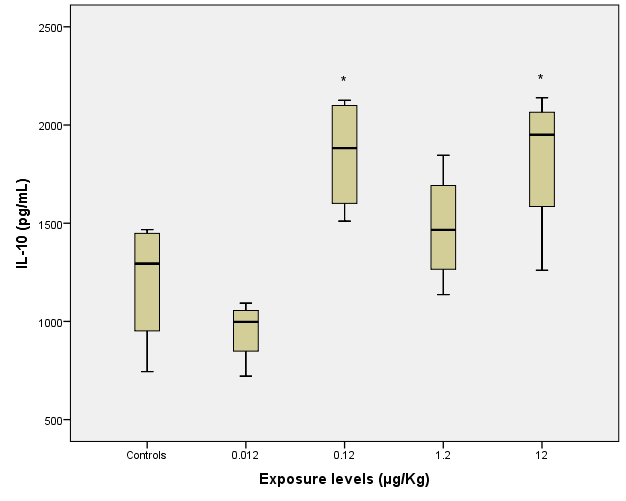


* Group mean significantly different from controls mean (p value < 0.05)

S1F Figure Serum levels of cytokine IL-12 of control rats and of four groups of female Wistar rats exposed to different levels of palladium nanoparticles.


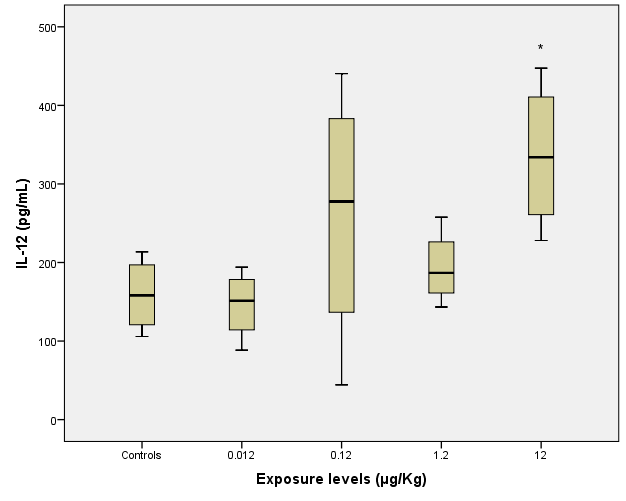


* Group mean significantly different from controls mean (p value < 0.05)

S1G Figure Serum levels of cytokine GM-CSF of control rats and of four groups of female Wistar rats exposed to different levels of palladium nanoparticles.


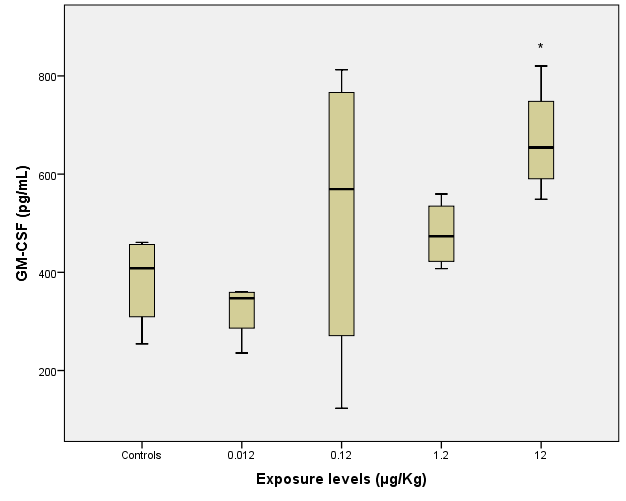


* Group mean significantly different from controls mean (p value < 0.05)

S1H Figure Serum levels of cytokine INF-γ of control rats and of four groups of female Wistar rats exposed to different levels of palladium nanoparticles.


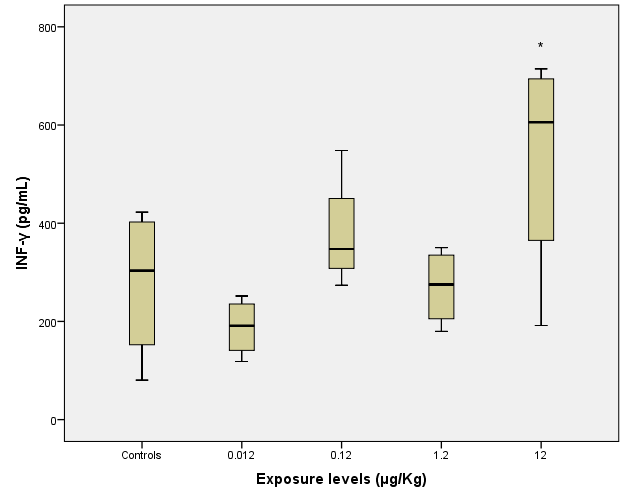


* Group mean significantly different from controls mean (p value < 0.05)

S1I Figure Serum levels of cytokine TNF-α of control rats and of four groups of female Wistar rats exposed to different levels of palladium nanoparticles.


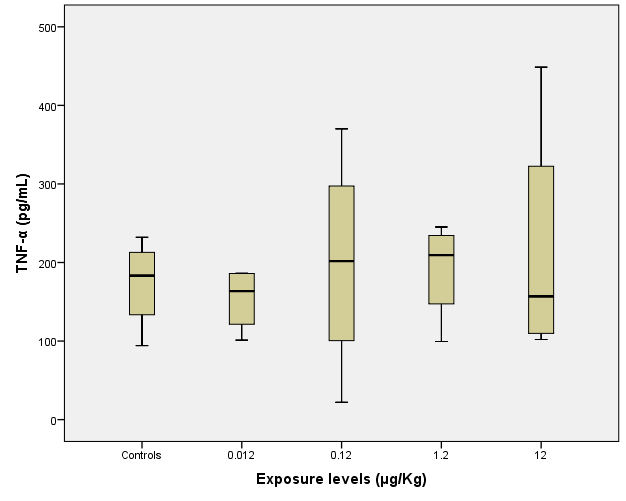

Supplement: S1 Fig — (DOCX) [file pone.0143801.s001.docx]
